# Resistance and resilience to Alzheimer's disease in Down syndrome

**DOI:** 10.1002/alz.70151

**Published:** 2025-04-28

**Authors:** Rory Boyle, Elouise A. Koops, Beau Ances, Elizabeth J. Andrews, Eider M. Arenaza‐Urquijo, Alexandre Benjanin, Adam M. Brickman, Rachel F. Buckley, Giulia S. Clas, Emmet Costello, Gillian T. Coughlan, Alexander C. Conley, Feng Deng, Daniele de Paula Faria, Natalie Edwards, Lisi Flores‐Aguilar, Juan Fortea, Ladan Ghazi Saidi, Elizabeth Head, Christy L. Hom, Katherine Koenig, Patrick Lao, Imre Lengyel, Yi‐Ju Li, Samantha Loi, David Loughrey, Eimear McGlinchey, Paul Newhouse, Lucía Pertierra, Prokopis C. Prokopiou, Qing Qi, Elisa de Paula França Resende, Jason Russell, Catherine E. Scanlon, Christoph Schneider, Stephanie A. Schultz, Mabel Seto, Sophia Shaka, Anja Soldan, Lídia Vaqué Alcázar, Yihe Weng, Jo Ellen Wilson, Shahid H. Zaman, Sára E. Zsadányi, Sigan Hartley

**Affiliations:** ^1^ Penn Frontotemporal Degeneration Center Perelman School of Medicine Richards Medical Laboratories University of Pennsylvania Philadelphia Pennsylvania USA; ^2^ Department of Radiology Massachusetts General Hospital, Harvard Medical School Boston Massachusetts USA; ^3^ Department of Neurology Center for Advanced Medicine Neuroscience Center Washington University in St. Louis St. Louis Missouri USA; ^4^ Department of Pathology and Laboratory Medicine University of California Irvine California USA; ^5^ Barcelona Institute for Global Health, Environment and Health Over the Life Course Programme, Climate, Air Pollution, Nature and Urban Health Programme Pompeu Fabra University Carrer de la Mercè Rosselló Barcelona Spain; ^6^ Sant Pau Memory Unit, Department of Neurology, Biomedical Research Institute Sant Pau Universitat Autònoma de Barcelona, Hospital de la Santa Creu i Sant Pau, Sant Antoni Maria Claret Barcelona Spain; ^7^ Center of Biomedical Investigation Network for Neurodegenerative Diseases Planta Madrid Spain; ^8^ Taub Institute for Research on Alzheimer's Disease and the Aging Brain G.H. Sergievsky Center Department of Neurology Columbia University Irving Medical Center New York New York USA; ^9^ Department of Neurology Massachusetts General Hospital and Harvard Medical School Boston Massachusetts USA; ^10^ Laboratory of Neurodegenerative Diseases Institute of Neurosciences Foundation for the Fight Against Childhood Neurological Diseases (LEN‐INEU‐Fleni‐CONICET), Fleni, Ciudad Autónoma de Buenos Aires Buenos Aires Argentina; ^11^ Academic Unit of Neurology Trinity Biomedical Sciences Institute, Trinity College Dublin Dublin Ireland; ^12^ Center for Cognitive Medicine Department of Psychiatry and Behavioral Sciences Vanderbilt University Medical Center Nashville Tennessee USA; ^13^ School of Psychology Shenzhen University Shenzhen China; ^14^ Laboratory of Nuclear Medicine (LIM43) Department of Radiology and Oncology, Faculdade de Medicina FMUSP Universidade de Sao Paulo Sao Paulo Sao Paulo Brazil; ^15^ Centro de Investigación Biomédica en Red de Enfermedades Neurodegenerativas. CIBERNED Barcelona Spain; ^16^ Barcelona Down Medical Center Fundació Catalana Síndrome de Down Barcelona Spain; ^17^ Department of Speech Language Pathology University of Nebraska at Kearney Kearney Nebraska USA; ^18^ Department of Pathology & Laboratory Medicine and Neurology University of California Irvine California USA; ^19^ Department of Psychiatry & Human Behavior University of California Irvine California USA; ^20^ Imaging Sciences, Diagnostics Institute Cleveland Clinic Cleveland Ohio USA; ^21^ The Wellcome‐Wolfson Institute for Experimental Medicine School of Medicine Dentistry and Biomedical Sciences Queen's University Belfast Belfast UK; ^22^ Department of Biostatistics and Bioinformatics Duke University School of Medicine, Duke University Medical Center Durham North Carolina USA; ^23^ Department of Psychiatry University of Melbourne; Neuropsychiatry Centre, Royal Melbourne Hospital Parkville Victoria Australia; ^24^ School of Nursing, Psychotherapy and Community Health Dublin City University Dublin Ireland; ^25^ Global Brain Health Institute Trinity College Institute of Neuroscience, Trinity College Dublin Dublin Ireland; ^26^ GBHI Memory and Aging Center University of California, San Francisco San Francisco California USA; ^27^ Trinity Centre for Ageing and Intellectual Disability Trinity College Dublin Dublin Ireland; ^28^ Geriatric Research, Education, and Clinical Center Veterans Affairs Tennessee Valley Health System Nashville Tennessee USA; ^29^ Cognitive Neurology, Neuropsychology and Neuropsychiatry Unit Buenos Aires Argentina; ^30^ Department of Radiology Massachusetts General Hospital and Harvard Medical School Boston Massachusetts USA; ^31^ Trinity College Institute of Neuroscience Trinity College Dublin Dublin Ireland; ^32^ Faculdade de Medicina Universidade Federal de Minas Gerais Belo Horizonte Brazil; ^33^ Department of Psychological and Brain Sciences Drexel University Philadelphia Pennsylvania USA; ^34^ Department of Neurology Inselspital and University of Bern Rosenbühlgasse Bern Switzerland; ^35^ Department of Neurology Brigham and Women's Hospital and Harvard Medical School Boston Massachusetts USA; ^36^ Department of Neurology University of California, Irvine Irvine California USA; ^37^ Department of Neurology Johns Hopkins University School of Medicine Baltimore Maryland USA; ^38^ Department of Psychiatry School of Medicine University of Cambridge Cambridge UK; ^39^ School of Human Ecology and Waisman Center, Nancy Nicholas Hall University of Wisconsin‐Madison Madison Wisconsin USA

**Keywords:** brain maintenance, brain reserve, cognitive reserve, cognitive resilience, dementia, trisomy 21

## Abstract

Due to the high prevalence of Alzheimer's disease (AD) in adults with Down syndrome (DS), trisomy 21 is now considered a genetic form of AD (DSAD). A better understanding of factors that can prevent or delay AD is vital to improve outcomes for adults with DS. In this narrative review, we apply AD and cognitive aging research frameworks to study resistance and resilience in DSAD. Given the variability in the timing of pathology and symptoms, we discuss the evidence supporting the role of genetic, biological, socio‐behavioral, lifestyle, and environmental factors in resistance and resilience to DSAD. We also consider how co‐occurring health conditions in DS may influence resistance and resilience, and how methods from AD research can be applied to DSAD. Ultimately, this framework aims to guide future research and translate findings into clinical interventions to improve outcomes in DSAD.

**Highlights:**

- Definitions of resistance and resilience in the genetic form of Alzheimer's disease (DSAD) are proposed for guiding the field.
- Variability in the timing of AD pathology and symptoms suggests the potential for resistance and resilience mechanisms in DSAD.
- Genetic, biological, socio‐behavioral, lifestyle, and environmental factors have the potential to build resistance or resilience in DSAD.
- Future research will require longitudinal and experimental designs, life course approaches, and large cohort studies.

## 1 INTRODUCTION

Down syndrome (DS) results from the full or partial triplication of chromosome 21 (chr21) or mosaicism. It is the leading known genetic cause of intellectual disability, with more than 5.8 million people worldwide having DS.^1^ The DS phenotype is characterized by mild to severe intellectual disability and several co‐occurring medical conditions, including congenital heart defects, gastrointestinal problems, immune disorders, hypothyroidism, sleep apnea, and vision and hearing impairment.^2^ Strikingly, individuals with DS have a 90% lifetime prevalence of Alzheimer's disease (AD),^3^ such that trisomy 21 is now seen as a genetic form of AD (DSAD) similar to autosomal dominant AD (ADAD), which is caused by mutations in the amyloid beta (Aβ) precursor protein (*APP*), presenilin 1 (*PSEN1*), or presenilin 2 (*PSEN2*) genes. Thus, AD has been identified as the key limitation to improving the lifespan for people with DS.^3^ Efforts to identify interventions that can delay or prevent AD are therefore of critical importance to the DS community.

In DS, the hallmark pathological features of AD emerge earlier in the lifespan relative to sporadic late‐onset AD (LOAD) in the neurotypical population, with Aβ plaques typically present in the 30s.^4^, ^5^, ^6^, ^7^ The early onset of AD pathology in DS is driven by the triplication of the *APP* gene located on chr21, which increases production of the Aβ peptide.^8^ Amyloid positivity is followed by intracellular neurofibrillary tangles^9^, ^10^ and finally by neurodegeneration, as evidenced by altered brain glucose metabolism, atrophy, and biomarkers of neuronal injury.^11^, ^12^ Similar to ADAD and LOAD, there is a long preclinical phase in DSAD, with clinical AD symptoms evident about 20 years following initial Aβ accumulation.^9^, ^13^

In recent years, the National Institutes of Health (NIH) has devoted more than $125 million in research funding to establish biomarkers of DSAD and launch trial‐ready DSAD studies (https://www.nih.gov/include‐project). Similar research efforts are being coordinated and funded across the globe. These efforts have led to large cohort studies (see Table 1), including the Alzheimer Biomarkers Consortium of Down syndrome (ABC‐DS), Trial Ready Cohort–Down Syndrome (TRC‐DS), London Down Syndrome Consortium (LonDownS), European Horizon 21 Consortium, and the Down Alzheimer Barcelona Neuroimaging Initiative (DABNI). Research from these cohort studies is quickly advancing science on the progression and timing of DSAD, information that is essential for designing AD clinical trials. Many AD clinical trials in the pipeline will directly target AD pathology (e.g., anti‐amyloid drugs). As with LOAD and ADAD, there are also efforts to identify protective lifestyle and biological factors that could be targeted in clinical trials as a means of delaying and/or preventing DSAD.

**TABLE 1 alz70151-tbl-0001:** Currently available datasets for investigating resilience and resistance to DSAD.

Study	*N*	Neuropsychology	Clinical	Neuropathology	PET	MRI	CSF	Genetics	Blood	EEG	Lifestyle, socio‐behavioral, environmental
ABC‐DS	550	X	X		X	X	X	X	X		X
DS‐BAI	120	X	X			X			X	X	
DABNI	1200	X	X		X	X	X	X	X	X	X
DSBC	304	X	X	X							
Horizon‐21^b^	1335	X	X					X	X		X
IDS‐TILDA	753	X	X								X
LonDownS	350	X	X					X	X	X	
NACC	500	X	X		X	X	X	X	X		X
Vitamin E Trial^a^	337	X	X								
Health system‐linked biobanks (e.g., PMBB, UKB, BioVU)	>1000	X	X		X	X	X	X	X		X

Abbreviations: ABC‐DS, Alzheimer Biomarkers Consortium—Down Syndrome (www.nia.nih.gov/research/abc‐ds); BioVU, Vanderbilt University Medical Center Biobank (https://victr.vumc.org/what‐is‐biovu/); CSF, cerebrospinal fluid; DABNI, Down Alzheimer Barcelona Neuroimaging Initiative (https://santpaumemoryunit.com/alzheimer‐down‐unit/dabni‐down‐alzheimer‐barcelona‐neuroimaging‐initiative/); DS‐BAI, Down Syndrome—Basque Alzheimer Initiative (https://doi.org/10.3390/jcm13041139); DSBC, Down Syndrome Biobank Consortium (https://medschool.cuanschutz.edu/neurosurgery/research‐and‐innovation/services/down‐syndrome‐biobank); IDS‐TILDA, Intellectual Disability Supplement to the Irish Longitudinal Study on Aging (https://idstilda.tcd.ie/); LonDownS, London Down Syndrome Consortium (www.ucl.ac.uk/london‐down‐syndrome‐consortium); MRI, magnetic resonance imaging; PET, positron emission tomography; NACC, National Alzheimer's Coordinating Center (https://naccdata.org); PET, Positron Emission Tomography; PMBB, Penn Medicine Biobank (https://pmbb.med.upenn.edu/data‐access/index.php); UKB: UK Biobank (https://www.ukbiobank.ac.uk/); Vitamin E Trial: (https://doi.org/10.1212/wnl.0000000000002714).

^a^ Denotes randomized controlled trial, whereas all other studies are observational studies. Reported Ns are approximate.

^b^ Horizon‐21 (https://horizon-21.org/) includes participants from England (LonDownS and the Cambridge Dementia in Down's Syndrome [DiDS] cohorts), Germany (AD21 study group, Munich), France (TriAL21 for Lejeune Institute, Paris), Spain (DABNI), and the Netherlands (the Rotterdam Down syndrome study).

Toward this latter goal, in 2023, two Professional Interest Area (PIA) groups of the Alzheimer's Association International Society to Advance Alzheimer's Research and Treatment (ISTAART)—the Reserve, Resilience, and Protective Factors PIA and the Down Syndrome and Alzheimer's Disease PIA—formed a working group to assess the current state of research on resistance and resilience to DSAD, the concepts of which are explained below. This working group consisted of researchers and clinicians with expertise related to resistance and resilience to AD in LOAD and ADAD, as well as experts in DSAD. The present article outlines the five aims proposed by this working group: (1) Establish a framework for understanding resistance and resilience to DSAD to guide future research; (2) review evidence on within‐population variability in the timing of DSAD pathology and symptomology; (3) evaluate the genetic, biological, socio‐behavioral, lifestyle, and environmental factors with the most promise to promote resistance and resilience to DSAD; (4) examine how health conditions that frequently co‐occur with DS may affect resistance and resilience to DSAD; and (5) identify key methodological considerations for studies on resilience and resistance to DSAD and establish a roadmap for future research.

## 2 FRAMEWORK OF RESILIENCE AND RESISTANCE TO DSAD

In 2019, a collaborative group of experts developed a framework to understand individual differences in cognitive aging and AD in the general population. This group, referred to as the “Collaboratory for Research Definitions on Reserve and Resilience in Cognitive Aging and Dementia” defined the term *resilience* as an umbrella term that encompasses any concept related to the capacity of the brain to maintain cognitive function with aging and disease.^14^ The group also provided consensus definitions for specific mechanisms hypothesized to underlie *resilience*, including *brain reserve*, *brain maintenance*, and *cognitive reserve*. More broadly, in AD research to date, two conceptually different mechanisms have been distinguished: *resistance* to AD pathology and *resilience* to the effects of AD pathology.^15^ This distinction allows researchers to distinguish between factors that may help halt or slow the development or progression of AD pathological processes (e.g., Aβ and tau) (“*resistance*”) versus factors that delay or slow processes downstream of Aβ and tau burden and ultimately reduce or delay the clinical expression of AD (“*resilience*”).^15^ *Resistance* refers to the idea that some individuals have no or lower‐than‐expected AD pathology despite elevated risk for AD, such as being a carrier of the apolipoprotein E (*APOE*) ε4 allele.^15^ *Resilience* has been operationalized as cognitive or functional performance that is better than expected at a given level of pathology,^15^ that is, the attenuation of the presence of AD pathology on cognitive performance. Within this framework, *resilience* mechanisms may include (1) having greater neurobiological capital prior to the development of AD‐related pathology (*brain reserve*); (2) greater ability to maintain brain structure and function over time in the presence of AD‐related pathology (*brain maintenance*); or (3) better adaptation of cognitive strategies that compensate for AD‐related changes (*cognitive reserve*). Thus, *resilience* is not viewed as operating through a single mechanism; nor is it viewed as only a response to age‐related changes or AD‐related pathology, as it can reflect individual differences in brain structure and function modified over the lifespan (e.g., through education, occupation).^16^

**RESEARCH IN CONTEXT**

1. **Systematic review**: We applied research frameworks from cognitive aging and Alzheimer's disease (AD) to develop operational definitions of resistance and resilience in genetic form of AD (DSAD), and we identified factors that may alter the timing of AD pathology or onset of dementia in DSAD based on existing theoretical and empirical evidence.
2. **Interpretation**: Given that in Down syndrome (DS) development of AD pathology is virtually universal, resistance to DSAD can be considered the absence or reduced levels of AD pathology relative to similar‐aged adults with DS. Resilience to DSAD can be considered as better‐than‐expected cognitive performance at a given level of AD pathology relative to adults with DS of a similar age and premorbid intellectual disability level.
3. **Future directions**: Longitudinal studies and experimental designs using methods specialized for the DS population are needed to identify factors contributing to resistance and resilience in DSAD. Specific mechanisms that lead to resistance and resilience may be identified using life‐course approaches, which will also enable the detection of critical periods in which these mechanisms are embedded.

**FIGURE 1:**
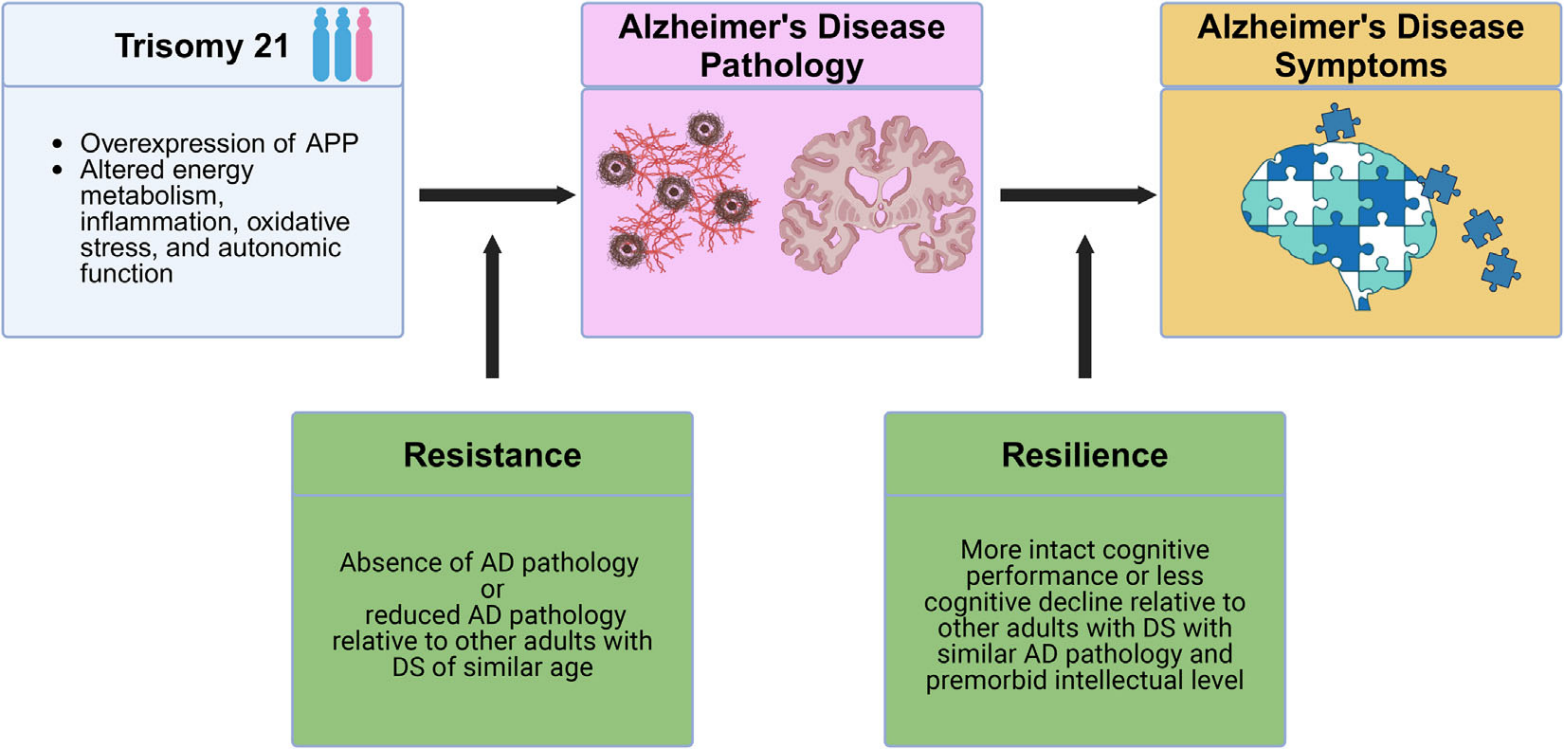
Resilience and resistance framework for DSAD. AD, Alzheimer's disease; APP, precursor protein; DS, Down syndrome.

These definitions of *resistance* [Table alz70151-tbl-0001] and *resilience* can be applied to DSAD with some modifications (Figure 1). In DSAD, the triplication of *APP* can be seen as conferring genetic risk for AD by driving Aβ accumulation, with other genes on chr21 compounding effects through altering energy metabolism, inflammation, oxidative stress, and autonomic functioning.^4^, ^13^ Evidence of *resistance* in DSAD should thus be broadened beyond the absence of AD pathology and also include reduced levels of AD pathology relative to other individuals with DS of similar age (given that individuals with DS are already at risk for AD due to trisomy 21). In contrast to LOAD, the genetic mechanisms driving DSAD confer greater predictability that individuals with DS will develop AD pathology, and the early age of onset reduces systemic aging‐related confounds; these differences may benefit the study of *resilience* factors, allowing better identification of key markers in DSAD compared to LOAD. However, given the varying lifelong levels of intellectual functioning among individuals with DS, *resilience* in DSAD can be operationalized as better‐than‐expected cognitive performance at a given level of AD pathology relative to other individuals with DS of similar age and premorbid intellectual disability level.

## 3 VARIABILITY IN THE TIMING OF PATHOLOGY AND SYMPTOMOLOGY IN DSAD

A prerequisite to the concept of resistance and resilience in DSAD is the presence of individual variability, specifically, evidence that the age at onset of AD pathology or rate of accumulation (resistance) and/or the age at onset of AD‐related cognitive impairment (resilience) varies within the DS population. In vivo, the pathological processes associated with AD can be measured[Table alz70151-tbl-0001] by biomarkers of aggregated Aβ, such as positron emission tomography (PET) Aβ‐PET or cerebrospinal fluid (CSF) Aβ42/Aβ40 protein ratios, and biomarkers of neurofibrillary tangles, such as tau‐PET and plasma tau.^15^ PET imaging in the ABC‐DS study demonstrates that abnormal Aβ accumulation becomes evident at ≈35 years of age and that abnormal tau deposition can be detected when individuals are in their 40s and 50s.^9^, ^17^, ^18^

There is, however, individual variability around the age of Aβ biomarker positivity (i.e., a threshold level suggestive of marked and broad accumulation). In a cross‐sectional study of 150 asymptomatic adults with DS in DABNI, CSF Aβ and phosphorylated tau‐181 (p‐tau181) negativity was still present for individuals in the oldest quartile (up to the age of 46.3 years).^19^ Similarly, in an ABC‐DS study, the mean age at onset of Aβ positivity on PET imaging was 46.4 years, with the youngest age of 33 years.^20^ Studies of longitudinal change in tau in individuals with DS are scarce. A study including 177 adults with DS from the ABC‐DS cohort reported that tau‐PET increased at the same rate across individuals following Aβ positivity onset,^9^ suggesting that variability in the timing of tau burden depends on previously established Aβ deposition. Of interest, this differs from what is observed in LOAD, where there is both a longer duration and greater heterogeneity in the association between the duration of Aβ positivity and elevated tau burden.

There is also evidence of variability in the timing of AD symptomology in DSAD. In a large meta‐analysis of published studies between 1968 and 2019 (*n* = 2695), the estimated age at DSAD dementia onset was 53.8 years, with a 95% confidence interval (CI) of 53.1–54.5 years.^3^ This estimated age at DSAD symptom onset was comparable to ADAD, preceding the average age at onset of symptoms in LOAD by 20 years. There was, however, substantial variability in the age at onset of DSAD dementia diagnosis, ranging from 35 to 74 years, with a marked subset of adults with DS over the age of 60 years remaining cognitively stable.

Evidence from neuropathological studies also shows variability in the level of AD‐related cognitive impairment based on age relative to the level of AD neuropathologic change among individuals with DS. In the general population, evidence for resilience includes postmortem studies of individuals with significant AD neuropathologic change who did not exhibit cognitive impairment or exhibited less‐than‐expected cognitive impairment prior to death.^21^ The Alzheimer's Disease Research Center at the University of California, Irvine (ADRC‐UCI), the DABNI cohort, and ABC‐DS have a unique collection of postmortem brain tissue from individuals with DS, which allows for this same evidence to be examined in individuals with DS. Among brain donors between 51 and 70 years of age at death with available clinical and neuropathological data, 13% of cases were non‐demented at their last clinical evaluation, despite intermediate or high AD neuropathologic change. This suggests that these individuals with DS were resilient to the effects of AD neuropathology on cognition (Flores Aguilar, unpublished data).

In summary, research to date demonstrates considerable variability in the age at onset of Aβ positivity in individuals with DS, with some showing lower levels of Aβ than expected given their age, a form of resistance to Aβ accumulation in DSAD. Evidence is less clear regarding resistance to tau, given that tau biomarker discovery is still in the early stage, and thus, there are fewer published studies on tau in DSAD. Findings to date suggest that the timing of tau accumulation following Aβ positivity is shorter and more homogeneous in DSAD relative to LOAD, but there may still be opportunities for resistance mechanisms to alter tau burden. There is also evidence for resilience in DSAD, given considerable variability in the timing of AD symptomology and age at dementia onset among individuals with similar levels of AD pathology. Postmortem studies also suggest that a subset of adults with DS with significant AD neuropathology did not develop clinical dementia.

## 4 GENETIC AND BIOLOGICAL FACTORS IN RESISTANCE AND RESILIENCE IN DSAD

### 4.1 Genetic factors

Several genetic factors are hypothesized to promote resistance and resilience to DSAD. Understanding their impact on the timing of DSAD can offer meaningful insight into underlying biological mechanisms that could be targeted in pharmaceutical interventions to build resistance or resilience to DSAD.

#### 4.1.1 *APP* gene

Triplication of the *APP* gene has been posited to be both necessary and sufficient for causing DSAD. This is corroborated by the observation of early‐onset AD in individuals without DS who have small internal duplications of chr21, leading to three copies of the *APP* gene (referred to as Dup‐APP).^22^ Conversely, individuals with DS with partial trisomy of chr21 that did not include an extra copy of the *APP* gene had little evidence of AD pathology and symptomology into their 70s.^23^ Thus, efforts to reduce the production of *APP* have the potential to provide resistance to DSAD. However, there is evidence that *APP* may not be the sole factor triggering AD pathogenesis in DSAD.^24^

#### 4.1.2 Mosaicism of chr21

Mosaicism occurs when somatic cells share different dosages of chr21 and accounts for 2%–4% of individuals with DS.^25^ Lacking the full dosage of overexpressed genes on chr21 may reduce risk for developing AD pathology,^26^ indeed, in two large DS cohorts (*n* = 357 and *n* = 468), lower plasma Aβ40 and Aβ42 concentrations were observed in adults with DS with mosaicism (vs full trisomy).^27^ Moreover, in the older of these two cohorts, the total and annual decline in cognitive performance was smaller, and the incidence and prevalence of dementia were lower among adults with DS with mosaicism (vs full trisomy).^27^ Similarly, in the ADRC‐UCI neuropathology cohort, there were three cases with mosaic DS. Among the mosaic DS cases (two female and one male, 48–55 years of age), one had dementia, one had mild cognitive impairment (MCI), and one was cognitively stable. Regarding tau pathology, the non‐demented case was categorized as Braak stage III, whereas DS cases of similar age tended to be Braak stage V or VI and demented. The DS case with dementia had tangle pathology consistent with Braak stage VI. Future studies should investigate if cells in the brain display mosaicism similar to that of other somatic cells, as this may alter the resistance effects of mosaicism.

Mosaicism can also be acquired over time.^28^ There is also recent epidemiological evidence to suggest that mosaicism may not be protective in DS.^29^ A case report of a person with trisomy 21 mosaicism showed early‐onset clinical dementia and significant AD neuropathology postmortem.^30^ Thus, AD neuropathology may be variable across the lifespan depending on the level of mosaicism.^28^ This is clearly an area of research that will benefit from further examination.

#### 4.1.3 Other chr21 genes

Other genes located on chr21 likely influence the progression of DSAD and may confer resistance or resilience. The region 21q22.11‐21q22.2 in the distal segment of the long arm of chr21 is described as the “DS critical region” due to its strong association with the DS phenotype, although the *APP* gene is not located in this region, and multiple regions may be relevant.^31^ Still, some genes within the DS critical region are linked to DSAD, such as *SOD1*, *DYRK1A*, *RUNX1*, and *ABCG1*.^31^, ^32^ In addition, a genome‐wide association study reported that the single nucleotide polymorphism (SNP) rs9808800 in the DS cell adhesion molecule (*DSCAM*) gene is associated with an earlier age‐at‐onset of AD dementia,^33^ suggesting a role for this gene in the clinical expression of DSAD. The *DSCAM* gene is also located in the DS critical region and is essential for neuronal wiring and motor learning. Moreover, variants found in the β‐secretase 2 (*BACE2*) gene (encoded in chr21), such as rs2252576, rs2837990, and rs7281733, are associated with an earlier age at onset of dementia in adults with DS. In contrast, the opposite was seen with the variants rs7510366 and rs6517664.^34^, ^35^ These results suggest that variants in *BACE2* may enhance a pathogenic role or mitigate a protective role of this protein.

#### 4.1.4 Non chr21 genes

Genetic variants in non‐chr21 genes are also implicated in modifying the age at onset of DSAD, such as SNPs in phosphatidylinositol‐binding clathrin assembly protein (PICALM) and variants in sortilin‐related receptor 1 (*SORL1*).^36^, ^37^ The missense variant rs605059 in the *HSD17B1* gene and the rs598126 variant in the *COASY* (2.2 (1.1, 4.4) gene on chr17 are associated with an earlier age at onset of AD dementia among women with DS.^38^ These two variants may also be associated with an earlier age at onset of AD in women without DS.^33^ Further research is needed to determine if these genes could serve as good targets for drug or therapeutic development to enhance resilience to DSAD.

#### 4.1.5 ApoE e4

The *APOE* ε4 allele is a risk factor for AD within and outside DS. Current evidence suggests that *APOE* ε4 shifts the age at which AD pathology begins to accumulate in a dose‐dependent manner, with ε4 homozygous individuals having the youngest age at onset of amyloid accumulation and positivity, followed by ε4 heterozygous individuals and then ε4 noncarriers.^39^ In the general population, nearly all individuals who are *APOE* ε4 homozygotes have elevated CSF Aβ in their 60s, and their lifetime risk for clinical AD is 60%–80%.^39^ There is mixed evidence on whether *APOE* ε4 influences DSAD. In 464 adults with DS from the Cambridge Dementia in Down's Syndrome (DiDS) cohort and DABNI, adults with DS who were *APOE* ε4 carriers had a lower CSF ratio of Aβ1‐42/Aβ1‐40 in young adulthood, earlier increases in amyloid PET and plasma p‐tau181 levels_,_ earlier reductions in cortical metabolism and hippocampal volume, and earlier memory decline than non‐carriers.^40^ Overall, adults with DS who were *APOE* ε4 carriers had an average age at AD dementia onset of 2 years (age 51) younger than those who were not *APOE* ε4 carriers (age 53),^40^ a finding also observed across various cohorts from the Horizon 21 European DS consortium.^41^ In contrast, *APOE* ε4 effects were not observed in the ABC‐DS cohort in regard to timing of amyloid^42^ or tau.^10^ More research is thus needed on *APOE* ε4 effects in DSAD and biological mechanisms driving any resistance or resilience effects. Evidence from an autopsy study of DS cases suggested that ApoE proteolysis generates an amino‐terminal fragment that accumulates within neurofibrillary tangles.^43^ Therapeutic interventions that reduce or remove these fragments may foster resistance to DSAD in *APOE* ε4 carriers.

### 4.2 Biological sex

The issue of biological sex differences in resilience and resistance to DSAD has not yet been studied comprehensively, although there are initial mixed findings.^23^, ^44^, ^45^ Some studies report no sex difference in timing or prevalence of DSAD,^44^ whereas others report increased risk in women at younger ages^23^ but greater risk of AD dementia in men after age 60.^45^ Several factors may explain sex‐specific resilience, including the contribution of hormones. Women with DS experience menopause 5–7 years earlier than the general population, and earlier age at menopause is associated with earlier onset of AD dementia in DS.^46^ In a study of 275 adults with DS, cognitively impaired women with DS (both MCI and AD dementia groups) showed elevated plasma total tau compared with cognitively stable women with DS, but this difference was not apparent among men, highlighting that women may bear greater loads of pathology despite having similar clinical presentation to men.^47^ It has also been reported that women with DS who were *APOE* ε4 carriers were diagnosed 3 years earlier than non‐carrier women, whereas this difference was not seen in men.^48^ Further work is needed to investigate biological sex as a factor in resistance and resilience, and continued reporting of sex disaggregated data will shed more light on this issue in DSAD.

### 4.3 Neuromodulatory system

Neuromodulators are a subclass of neurotransmitters that are released by neurons in subcortical nuclei diffusely and can affect multiple cell types and brain regions. Neuromodulators modulate neuronal responses to other neurotransmitters, for example, by influencing the activity of the autonomic nervous system, which may play a key role in resilience to DSAD. Here we review evidence suggesting important roles of the noradrenergic and cholinergic systems, particularly in resistance and resilience in DSAD.

#### 4.3.1 Noradrenergic system

The locus coeruleus, the primary noradrenergic nucleus of the brain, has an important role in memory formation and arousal, and greater structural integrity and novelty‐related activation of the locus coeruleus may be protective against the downstream effects of AD pathology on cognition.^49^ This finding suggests that the locus coeruleus is important for resilience, and while the relevance of locus coeruleus structural and functional alterations on cognition in individuals with DS has not yet been established, reduced serum levels of 3‐Methoxy‐4‐hydroxyphenylglycol (MPHG), a noradrenergic compound, have been reported in adults with DSAD versus non‐demented adults with DS and adults without DS.^50^

#### 4.3.2 Cholinergic system

The cortical cholinergic system, critical for learning, memory, and attention,^51^ is affected in AD and DS. Observed deficits in cholinergic function have been associated with the progressive degeneration of basal forebrain cholinergic neurons, the primary cholinergic output of the central nervous system.^52^ In DS, degeneration of the nucleus basalis of Meynert neurons (located in the basal forebrain) begins in early adulthood, preceding overt AD symptoms,^52^ and corresponds to the timing of early AD biomarker changes.^53^ The relevance of interventions in the cholinergic system in DSAD has been studied using the Ts65Dn mouse model of DSAD, where maternal dietary choline supplementation reduced the degeneration of basal forebrain cholinergic neurons and improved spatial memory function in the offspring.^54^ In human adults with DSAD, targeting the cholinergic system with cholinesterase inhibitor treatment has improved cognitive endpoints.^55^ In addition, novel cholinergic therapies, such as positive allosteric modulators of cholinergic receptors, are under active investigation.^56^ These findings highlight that cholinergic therapies and nutritional supplementation may promote a more resilient cholinergic system, potentially sparing cognitive function and protecting against AD pathology in individuals with DS later in life.

### 4.4 Summary

To date, the field has identified evidence implicating several genetic and biological factors in resistance and resilience to DSAD. The partial trisomy and mosaicism of chr21 are associated with less‐than‐expected AD pathology (although there are some variable associations with protection) and may confer resistance to DSAD. Although it might be debated whether an individual with reduced production of Aβ pathology from birth due to differences in their genetic makeup (i.e., partial trisomy and mosaicism) is truly resistant, our broad definition of resistance in DSAD considers this so. Aside from *APP*, other chr21 genes may attenuate resistance and resilience, given associations with earlier age at onset of AD in DS. *APOE* ε4 is also associated with earlier age at onset AD dementia in DS, but further research is needed to gain a clearer picture of the relevance of other non‐Chr21 genes. Future research on transcriptomic and proteomic factors will help our understanding of their relevance to resistance and resilience in DSAD. Resilience may differ across the biological sexes, but given the mixed findings to date, sex‐stratified analyses are needed to further ascertain the role of sex‐specific factors. Although the noradrenergic neuromodulatory system has a role in resilience to AD, its role in resilience to DSAD has not yet been studied. The beneficial effects of cholinesterase inhibitors on cognition in DSAD suggest that the cholinergic system could promote resilience to DSAD.

## 5 SOCIO‐BEHAVIORAL, LIFESTYLE, AND ENVIRONMENTAL FACTORS IN RESISTANCE AND RESILIENCE TO DSAD

Socio‐behavioral and environmental factors have also been posited to be associated with resistance and, most frequently, resilience to DSAD, as documented in the broader AD literature. The most promising factors from research to date are education and occupation, leisure activities, and physical activity. Research on these factors has stemmed mainly from cross‐sectional or observational studies and often focused on resilience in terms of better‐than‐expected cognitive performance for age.

### 5.1 Premorbid intellectual disability level

The DS population displays a considerable range in intelligent quotient (IQ), with about 75% having mild or moderate intellectual disability and 15%–25% with severe to profound intellectual disability.^57^ It is posited that a higher IQ confers greater ability to recruit alternate neural networks or use existing networks more efficiently to cope with early AD‐related pathology. In DS, variability in IQ is influenced by the type of trisomy.^58^ However, similar age trajectories in the accumulation of PET Aβ and tau in the onset, and rate, of cognitive decline among individuals with DS with varying levels of premorbid intellectual disability have been reported.^59^ Similarly, no differences in the average age of individuals with a clinical status of MCI or AD dementia were found across individuals with mild, moderate, or severe/profound intellectual disability.^59^ Thus, research to date does not suggest that the level of intellectual disability in and of itself serves as a resilience or resistance mechanism for DSAD. However, it is important to note that floor effects often occur on standardized IQ tests with individuals with DS, which can make it difficult to capture differences in IQ among people with IQs <40.

### 5.2 Employment and education

The potential contributions of education and occupational complexity (i.e., the extent to which one's job requires problem solving, critical thinking, and perspective taking) have been studied in DSAD. In one cross‐sectional study of 56 adults with DS ages 25–58 years (and thus already accumulating Aβ plaques), those engaged in more (vs less) complex employment had less cognitive decline across 16–20 months when controlling for age, intellectual disability level, and hours spent in employment.^60^ Another previous study of 35 adults with DS, ages 29–67 years, reported that higher education and employment levels (on a scale of no employment to full‐time employment in the community) were associated with better cognitive functioning.^61^ A promising development is that in many countries across the globe, adults with DS are increasingly engaging in employment,^62^ and there are now college programs geared toward adults with intellectual disability (see https://downsyndrome.ie/higher‐education/). This positive trend should be continued and further expanded, given that participation in education and employment may be a vital way to promote resilience in DSAD.

### 5.3 Leisure activities

Fewer studies have examined the role of cognitive and social leisure activity engagement in DSAD. In a study of 65 adults with DS (ages 30–53 years), the level of engagement in leisure activities at baseline was not related to baseline Aβ‐PET burden or rate of change in Aβ‐PET levels over 3 years,^63^ suggesting that leisure activity engagement does not provide resistance to Aβ accumulation. However, *social* activity engagement moderated the association between change in Aβ‐PET and decline in episodic memory performance across the 3‐year study period, indicating a potential resilience effect of leisure in DSAD.

### 5.4 Physical activity

Adults with DS engage in less physical activity than the general population,^64^ likely due in part to a combination of hypotonia, low muscle strength, impaired autonomic functioning, and higher levels of obesity.^65^ Nevertheless, in adults with DS, the time spent in moderate to vigorous physical activity has been positively associated with cognitive performance when controlling for age and premorbid intellectual disability level.^66^ Similarly, in a study that followed 214 participants for 12 months, engagement in greater moderate to vigorous physical activity at baseline was associated with a 62% reduced risk of decline in memory at 12 months,^67^ indicating that physical activity may support resilience. However, physical activity has not been linked directly to resistance, as null associations have been reported with hippocampal volume^68^ or longitudinal accumulation of Aβ‐PET.^63^

The mechanisms through which physical activity confers resistance or resilience to DSAD are unclear. Higher physical activity in adults with DS has been associated with reduced risk of obstructive sleep apnea, endocrine/metabolic conditions, and cardiovascular disease.^69^ Thus, physical activity may reduce the risk of DSAD by lowering the risk of co‐occurring health conditions. Greater physical activity has also been associated with better white matter microstructural integrity in adults with DS,^66^ which may thereby contribute to brain reserve.

### 5.5 Stressors, discrimination, and stigma

The impact of discrimination and stigma on resistance and resilience to DSAD requires consideration. Compared to the general population, individuals with DS face ongoing challenges in equitable access to health care and social care services.^70^ Individuals with disabilities are also at higher risk of maltreatment and victimization than individuals without disabilities.^71^ Such experiences, in addition to challenges in accessing services, may negatively affect physical and mental health and increase the levels of stress experienced by individuals with DSAD, which may attenuate resistance and resilience to AD. Biologically, chronic stress can drive systemic inflammation and vascular disease,^72^ two pathways involved in AD pathogenesis.^73^ Outside of DS, depression and social isolation have been identified as modifiable risk factors for AD.^74^ The shift away from institutionalized care over the past few decades toward greater social integration may contribute to better health outcomes for adults with DS above and beyond improvements in health care.^3^

Outside of DS, inequities in the social and structural determinants of health have been noted to give rise to racial disparities in AD dementia.^75^ In the United States, a higher risk of MCI and dementia has been reported in Hispanic and Black adults compared to White adults,^76^ and more rapid cognitive decline has been observed in older adults who were born in states with higher levels of structural and socioeconomic racism,^77^ suggesting that racial and ethnic disparities influence resilience. These disparities may be even more pronounced within the DS population.^78^ Approximately 80% of people with disabilities reside in low‐ and middle‐income countries.^79^ In high‐income countries, differences in wealth trajectories between parents with and without children with DS have been documented.^80^ Adverse outcomes in DS have broadly been linked to lower socioeconomic conditions.^81^ However, there is a lack of data regarding how reducing these disparities may improve resistance or resilience in DSAD.

### 5.6 Summary

Overall, cognitively stimulating activities related to education, employment activities, and leisure activities appear to have promise for increasing resilience to DSAD, albeit evidence to date is based primarily on cross‐sectional findings of better‐than‐expected cognitive performance given an individual's age as opposed to levels of AD biomarkers. There is also evidence that physical activity provides resilience as measured by better‐than‐expected cognitive performance at a given age or as increases in cognitive performance following intervention. Evidence to date suggests that the benefit of physical activity may not be directly related to AD pathophysiological processes (i.e., resistance) but may alter other aspects of brain functioning (e.g., white matter impairment and reduced co‐occurring health conditions) in ways that allow individuals with DS to tolerate early AD pathology for longer (i.e., resilience). Moving forward, it is important for the field to examine how social and structural determinants of health, including societal views of race, ethnicity, socioeconomic status, and disability, alter resistance and resilience to DSAD. Such efforts may also provide insights for designing targeted interventions and policy changes to improve access to medical care, educational and occupational opportunities, leisure activities, and physical activities. Future work could also consider how coping strategies, social support, and positive life events could provide resilience *by* buffering against the negative effects of stressors on biological processes that may contribute to DSAD.

## 6 TARGETING CO‐OCCURRING HEALTH CONDITIONS TO BUILD RESISTANCE AND RESILIENCE IN DSAD

Trisomy 21 is associated with a host of co‐occurring health and neurobiological processes. Efforts to target these conditions and the underlying biological processes may also offer meaningful pathways for building resistance and resilience to DSAD.

### 6.1 Late‐onset seizures

Late‐onset seizures are noted in LOAD, ADAD, and DSAD,^82^ which may be a result of the toxic accumulation of Aβ triggering synaptic degeneration, circuit remodeling, and abnormal synchronization of neuronal networks.^83^ The prevalence of late‐onset myoclonic epilepsy in DS (LOMEDS), characterized by cortical myoclonus and generalized tonic–clonic seizures, has been reported to be as high as 56% to 80%.^84^ These seizures could lead to additional neurologic insults and accelerate cognitive decline.^82^ Therefore, the early identification and treatment of LOMEDS may increase resilience by slowing the rate of symptomatic decline.

### 6.2 Cardiometabolic disease and obesity

Trisomy 21 is associated with alterations in gene expression that impact metabolism and metabolic health. For example, many of the genes on chr21 (e.g., *S100β*, *SOD1, PIGP*) influence pathways involved in inflammation,^85^ oxidative stress response,^86^ as well as lipid and energy metabolism.^87^ The downstream physiological effects of dysregulated metabolism are thought to alter energy intake and expenditure in ways that lead to obesity and increase risk for other co‐occurring conditions such as obstructive sleep apnea and cardiometabolic disease.^88^ Metabolic dysfunction, obesity, and diabetes type 2 have also been theorized to contribute to DSAD through effects on insulin resistance and glucose dysregulation, oxidative stress, and vascular damage that may increase Aβ plaques and hyperphosphorylated tau.^89^ However, research investigating these effects is limited in DS and thus it is not clear if efforts to improve metabolic health, including reducing obesity and type 2 diabetes, build resistance to DSAD.

### 6.3 Immune dysfunction and inflammatory processes

Neuroinflammation plays a role in AD pathogenesis and neurodegeneration, and individuals with DS have a uniquely elevated inflammatory profile that persists across the lifespan. This profile manifests as highly active and morphologically distinct astrocytes and microglia and increased levels of inflammatory cytokines,^85^ related to systemic and central nervous system inflammation. Co‐occurring health conditions such as periodontitis^90^ can also contribute to low‐grade chronic inflammation. Immune system dysregulation and neuroinflammation have long been posited to play a role in AD pathogenesis and neurodegeneration outside of DS.^91^ Further research is needed to investigate the possibility that prevention and treatment of autoimmune and inflammatory processes might contribute to resistance to AD pathology in DSAD. Similar to in LOAD, in adults with DS, plasma levels of glial fibrillary acidic protein (GFAP), a marker of reactive astrocytosis, differentiate between those with and without AD dementia, correlating strongly with Aβ pathology, neurodegeneration, and AD clinical progression.^92^ Furthermore, the astrocyte‐associated protein, *S100β*, is also on chr21.

Although an exacerbated inflammatory response has been partially attributed to the triplication of a range of immune‐related genes located on chr21,^93^ synergism between inflammatory processes and the brain's vasculature may contribute to the inflammatory profile in DS.^94^ Magnetic resonance imaging (MRI) markers of cerebrovascular disease are associated with proteomic patterns reflective of inflammation earlier in the disease and patterns reflective of neurodegeneration later in the disease.^95^ Furthermore, cerebrovascular disease may promote tau pathology through astrocytic pathways in the preclinical stages of DSAD,^96^ suggesting that neuroinflammation, and potentially its biological interaction with vascular pathology, could be a meaningful target for building resistance to DSAD.

### 6.4 Cerebrovascular disease

Cerebrovascular disease is highly prevalent in adults with DS,^97^, ^98^ who show white matter hyperintensities, enlarged perivascular spaces, and infarcts on MRI as early as in their 40s.^99^ Moreover, the presence of cerebrovascular pathology increases in line with the severity of cognitive impairment in DSAD.^99^ Postmortem analyses demonstrate significantly lower microvessel density in DS cases than in non‐DS cases.^100^ Although atherosclerosis and arteriolosclerosis are rare in postmortem DS cases, cerebral amyloid angiopathy, that is, the deposition of Aβ in small vessels, is observed more frequently in DS cases compared to AD and control cases,^101^ which is unsurprising given the overproduction of Aβ in DS. In DS, the severity of cerebral amyloid angiopathy is associated with microbleeds.^102^ The co‐occurring presence of cerebrovascular pathology in DSAD may thus deplete resilience to AD pathology. Postmortem examinations of cognitively unimpaired individuals with DS can provide some insight into the detrimental effect of co‐occurring cerebrovascular pathology on resilience to AD. For instance, in the 90+ Study, a non‐DS cohort, resilient cases (cognitively unimpaired despite pathological diagnoses of AD) showed significantly fewer non‐AD pathologies at postmortem than AD cases.^21^

### 6.5 Sensory impairments

Visual impairments, such as nystagmus, strabismus, keratoconus, amblyopia, cataracts, and refractive errors, are common in adults with DS,^103^ and retinal changes may underpin some of the visual impairments in DS. However, it is not yet clear if visual impairments influence DSAD pathology or accelerate decline. Outside of DS, vision impairments have been associated with an increased risk of AD.^104^ Adults with DS are also at risk for hearing impairments, including conductive, sensorineural, and mixed types.^105^ Central auditory processing abnormalities in individuals with DS are reported, potentially in relation to the degeneration of the cholinergic system, indicating possible AD neuropathological overlap.^106^ Hearing loss is considered a modifiable risk factor for dementia outside of DS, and addressing hearing loss, thereby maintaining access to environmental cognitive stimulation, may promote resilience to DSAD.^74^

### 6.6 Disrupted sleep and obstructive sleep apnea

Sleep disruptions, common in individuals with DS,^101^ have been associated with higher PET Aβ and lower cognitive performance in 47 adults with DS.^107^ Moreover, more disrupted sleep is observed in adults with DS and MCI compared to cognitively stable adults with DS.^107^ In another study of 116 adults with DS (36% with MCI or AD), an obstructive sleep apnea diagnosis was associated with higher cortical PET Aβ and greater white matter hyperintensity volume in the frontal and temporal lobes.^108^ Impaired white matter microstructural integrity has been identified as potentially driving the connection between disrupted sleep and obstructive sleep apnea with DSAD. Given the high prevalence of sleep disorders in the DS population, addressing sleep disturbances and reducing obstructive sleep apnea may provide avenues for building both resilience and resistance to DSAD.

### 6.7 Summary

As we have reviewed, people with DS can experience several co‐occurring health conditions and targeting these conditions may promote resistance and resilience to DSAD. Although late‐onset seizures, cardiometabolic disease, and obesity could theoretically affect both resistance and resilience to DSAD, more research is needed to determine whether addressing these conditions will meaningfully promote resistance and resilience. Immune dysfunction and neuroinflammation and the interaction of neuroinflammation with cerebrovascular disease are likely important factors affecting resistance to DSAD and addressing cerebrovascular disease may improve resilience. Correction of sensory impairments may promote resistance and resilience, but has not yet been well‐studied in DSAD. In contrast, findings from several studies have implicated sleep disruptions and sleep disorders in resistance to DSAD. These is not an exhaustive list of co‐occurring conditions, but we have described some related to differential outcomes in DSAD and mechanisms through which they might influence resistance and resilience. Other co‐occurring health conditions in DS should be studied further to understand their influence on resistance and resilience. For example, musculoskeletal problems^109^ may affect engagement in physical activity or activities requiring travel, or hypothyroidism^110^ may impair cerebral blood flow and glucose metabolism.^111^ Continued work addressing the role of co‐occurring health conditions will help to understand better the mechanisms through which they might affect resistance and resilience to DSAD and may help to identify interventions that address comorbidities while also promoting resistance and resilience.

## 7 METHODOLOGICAL CONSIDERATIONS FOR STUDYING RESISTANCE AND RESILIENCE IN DSAD

### 7.1 PET imaging

PET imaging has been applied to study biomarker changes and A/T/(N) staging in DSAD and offers a framework for understanding resistance and resilience to DSAD. In line with findings from LOAD, clinical AD symptomatology was more closely associated with tau than Aβ,^18^ emphasizing the importance of tau‐PET for studying resilience in later stages of the DSAD continuum. Moreover, neuroinflammation PET imaging may provide even earlier markers indicating pathological change preceding changes in the other biomarkers.^112^ PET imaging can also inform A/T/(N) staging in DS, a useful framework to explore resilience and resistance at different stages of the AD continuum. For instance, in a sample of 162 adults with an age range of 25 to 61 years (38.84 ± 8.41), 69.8% were A–/T–/(N) –, 11.1% were A+/T–/(N) –, 5.6% were A+/T+/(N) –, and 9.3% were A+/T+/(N)+.^18^ PET imaging concerning A/T/(N) staging in DSAD warrants more investigation, primarily to better determine thresholds for this population. It may also be an essential tool in AD clinical trials for DSAD, as it is for the general population.

### 7.2 Structural imaging

Structural imaging studies have indicated that there is evidence of divergent structural connectivity in DS individuals^113^ and, importantly, have demonstrated lower total intracranial volume (TIV) in DS individuals compared to the general population.^114^ TIV has been used as a proxy for brain reserve^115^ and is often used to account for between‐subject variability in total and regional brain volume related to variation in head size. Thus, care is needed when assessing and interpreting brain substrates for resilience and resistance in DS, especially when using volumes adjusted for TIV and/or comparing results to the general population. In this regard, surface metrics that do not require TIV adjustment, microstructural properties derived from diffusion imaging, and studies using longitudinal within‐subject brain changes might be useful to overcome these issues.

Generally, as discussed, there are some challenges in the use of these imaging modalities in DS, which may be further exacerbated due to a higher likelihood of motion artifacts.^116^ However, implementing population‐specific approaches for data acquisition and processing^117^ and improved quality control of MRI data can help to mitigate these issues. The structural specificities in DS can also affect the suitability of some standard neuroimaging preprocessing pipelines, which have been developed in adults without DS. Developing DS‐specific standard anatomic templates^118^ and atlases might help uncover neural substrates of resilience and resistance in DS.

### 7.3 Functional imaging in DS

Abnormal functional connectivity in the default mode network (DMN)^119^ has been reported in DS (e.g., reduced strength of connections to posterior cingulate and anterior cingulate) and has been associated with the presence of AD neuropathology^120^ and with clinical progression to AD dementia in the DS population.^121^ Together, these findings suggest that intact functional connectivity of the DMN may contribute to both resilience and resistance to AD in DS. Future studies might obtain insights into functionally relevant substrates of resilience in DSAD by investigating whether functional connectivity of networks, such as the DMN or the frontoparietal control network, modify the relationship between AD pathology and cognitive decline, as reported in non‐DS populations.^122^ Measuring functional activation in adults with DS during episodic memory and executive tasks in the context of AD biomarkers could also provide insights into functionally relevant substrates of resilience in DSAD.

### 7.4 Currently available data

In Table 1, we have summarized publicly available datasets that include robust data from existing DS cohort studies that could be leveraged to shed new light on resilience and resistance to DSAD.

## 8 SUMMARY AND ROADMAP FOR STUDYING RESISTANCE AND RESILIENCE IN DSAD

There have been significant advances in understanding the natural history of DSAD. However, there are critical gaps in our current understanding of resilience and resistance factors in DSAD. Determining factors that delay AD pathology (i.e., resistance) and/or protect against downstream cognitive decline (i.e., resilience) in DSAD has important clinical and research implications, including driving pharmaceutical, lifestyle, or policy interventions to delay disease progression and/or prolong functioning. Moreover, in DSAD clinical trials, efforts to take into account resistance and resilience factors (e.g., presence of certain co‐occurring health conditions) could improve the sensitivity to treatment effects.^123^ Furthermore, the possibility of targeting biological resistance and resilience factors, including the cholinergic system or genetic factors, via novel therapeutics should be explored. Future results from postmortem studies from well‐characterized cohorts and multimodal datasets, as identified in Table 1, will enrich and deepen our knowledge of resilience and resistance to DSAD. Increased emphasis on data sharing and collaborations will give researchers greater access to large, robust datasets in the coming years.

Four themes emerge in thinking about advancing the understanding of resistance and resilience to DSAD over the next 5 years. First, there is a critical need for longitudinal studies and experimental designs (e.g., clinical trials of physical activity interventions), as most evidence for resistance and resilience to DSAD to date is based on cross‐sectional or observational studies. Second, the development of DS population‐specific tools for assessing lifestyle, socio‐behavioral, and environmental factors is needed to move the field forward, as many of the tools developed for the general population (e.g., education level or household income) may be irrelevant or less sensitive in assessing the variables most salient for people with DS (e.g., social inclusion and disability‐related discrimination). Third, a promising avenue for future research may be to profile “resistant” or “resilient” individuals to understand better the factors that may differentiate them from their peers. For example, such research could identify adults with DS in their 50s without elevated Aβ or those who are in their mid‐60s without AD dementia and leverage statistical approaches to determine what genetic, biofluid, co‐occurring neurological, or lifestyle factors (past and present) distinguish these individuals from others. Fourth, there is a need for life course approaches aimed at understanding how mechanisms that foster resistance or resilience are built over time to protect against DSAD. Indeed, life course approaches may be better suited for capturing the gradual accumulation of brain and cognitive reserve that stems from multiple resilience factors that build on one another over time. Long‐term longitudinal studies that observe individuals with DS across childhood and into adulthood may also identify resistance and resilience mechanisms that occur in early life (e.g., early life events or medical interventions for early life conditions such as congenital heart deficits), which are often ignored in aging research and can also help to identify if there are critical periods for the development of resistance and resilience in DSAD. Fifth, it is important for the field to continue to refine its definitions of resistance and resilience as new discoveries come to light. Specifically, the field will need to consider to what level of deviation from the mean pattern signifies resistance (e.g., delay of Aβ positivity by 3 or 10 years) and resilience (e.g., delay of AD dementia by 5 or 15 years), as this may differ from that of LOAD, in which there is not the same strong genetic determinant. Further revisions of definitions may also consider the need for conceptual separations between modifiable and non‐modifiable resistance and resilience factors.

In conclusion, the application of the framework of resistance and resilience to DSAD as outlined here provides an opportunity for researchers to further explore the genetic, biological, socio‐behavioral, lifestyle, and environmental factors involved in resistance and resilience. These future explorations, along with a better understanding of the role of co‐occurring health conditions in DSAD and improvements in imaging and data availability provide a clear path forward to improving our understanding of resistance and resilience in DSAD and to ultimately translating this knowledge into improved outcomes for people with DS.

## CONFLICT OF INTEREST STATEMENT

J.F. received fees for service on advisory boards, committees, or speaker honoraria from AC Immune, Adamed, Alzheon, Biogen, Eisai, Esteve, Fujirebio, Ionis, Laboratorios Carnot, Life Molecular Imaging, Lilly, Lundbeck, Perha, and Roche. O.B., D.A., A.L. and J.F. report holding a patent for markers of synaptopathy in neurodegenerative disease (licensed to Adx, EPI8382175.0). L.G.S. received speaker honoraria from Nebraska Speech and Language Hearing Association, Eleanor M. Saffran Center for Cognitive Neuroscience, Temple University, and University of Quebec at Traois Riviers. S.L.H. received fees from Ionis and Alzheon. Author disclosures are available in the Supporting Information.

## Supporting information



Supporting Information
