# Correction to “Resistance and resilience to Alzheimer's disease in Down syndrome”

**DOI:** 10.1002/alz.70302

**Published:** 2025-05-19

Boyle R, Koops EA, Ances B, et al. Resistance and resilience to Alzheimer's disease in Down syndrome. *Alzheimers Dement*. 2025; 21:e70151. https://doi.org/10.1002/alz.70151

In the author list, Alexandre Bejanin's surname was listed as “Alexandre Benjanin.” This was incorrect and should have read “Alexandre Bejanin.”

We apologize for this error.

